# Identification of a Highly Antigenic Linear B Cell Epitope within *Plasmodium vivax* Apical Membrane Antigen 1 (AMA-1)

**DOI:** 10.1371/journal.pone.0021289

**Published:** 2011-06-21

**Authors:** Lilian Lacerda Bueno, Francisco Pereira Lobo, Cristiane Guimarães Morais, Luíza Carvalho Mourão, Ricardo Andrez Machado de Ávila, Irene Silva Soares, Cor Jesus Fontes, Marcus Vinícius Lacerda, Carlos Chavez Olórtegui, Daniella Castanheira Bartholomeu, Ricardo Toshio Fujiwara, Érika Martins Braga

**Affiliations:** 1 Departamento de Parasitologia, Instituto de Ciências Biológicas, Universidade Federal de Minas Gerais, Belo Horizonte, Brazil; 2 Departamento de Bioquímica e Imunologia, Instituto de Ciências Biológicas, Universidade Federal de Minas Gerais, Belo Horizonte, Brazil; 3 Departamento de Análises Clínicas e Toxicológicas, Universidade de São Paulo, São Paulo, Brazil; 4 Departamento de Clínica Médica, Universidade Federal de Mato Grosso, Cuiabá, Brazil; 5 Fundação de Medicina Tropical Dr. Heitor Vieira Dourado, Manaus, Brazil; 6 Laboratório de Imunologia Celular e Molecular, Instituto René Rachou, FIOCRUZ, Belo Horizonte, Brazil; 7 Instituto Nacional de Ciência e Tecnologia de Vacinas (INCTV), Belo Horizonte, Brazil; 8 Instituto Nacional de Ciência e Tecnologia em Doenças Tropicais (INCT-DT), Salvador, Brazil; Agency for Science, Technology and Research (A*STAR), Singapore

## Abstract

Apical membrane antigen 1 (AMA-1) is considered to be a major candidate antigen for a malaria vaccine. Previous immunoepidemiological studies of naturally acquired immunity to *Plasmodium vivax* AMA-1 (PvAMA-1) have shown a higher prevalence of specific antibodies to domain II (DII) of AMA-1. In the present study, we confirmed that specific antibody responses from naturally infected individuals were highly reactive to both full-length AMA-1 and DII. Also, we demonstrated a strong association between AMA-1 and DII IgG and IgG subclass responses. We analyzed the primary sequence of PvAMA-1 for B cell linear epitopes co-occurring with intrinsically unstructured/disordered regions (IURs). The B cell epitope comprising the amino acid sequence 290–307 of PvAMA-1 (SASDQPTQYEEEMTDYQK), with the highest prediction scores, was identified in domain II and further selected for chemical synthesis and immunological testing. The antigenicity of the synthetic peptide was identified by serological analysis using sera from *P. vivax*-infected individuals who were knowingly reactive to the PvAMA-1 ectodomain only, domain II only, or reactive to both antigens. Although the synthetic peptide was recognized by all serum samples specific to domain II, serum with reactivity only to the full-length protein presented 58.3% positivity. Moreover, IgG reactivity against PvAMA-1 and domain II after depletion of specific synthetic peptide antibodies was reduced by 18% and 33% (P = 0.0001 for both), respectively. These results suggest that the linear epitope SASDQPTQYEEEMTDYQK is highly antigenic during natural human infections and is an important antigenic region of the domain II of PvAMA-1, suggesting its possible future use in pre-clinical studies.

## Introduction

Malaria is one of the most debilitating parasitic diseases of humans, with an estimated 225 million clinical cases and 781,000 deaths per year [1]. Of the species that infect humans, *Plasmodium vivax* and *Plasmodium falciparum* are the two most important human malaria parasites. Although deaths by *P. vivax* are rare compared to the *P. falciparum*, *P. vivax*-induced malaria has an enormous socioeconomic impact [2] and is now recognized as a cause of severe and fatal malaria [3]. There are an increasing number of publications reporting severe disease, including respiratory distress, severe anemia and coma, as a result of *P. vivax* infection [4], [5]. Considering the emergence of chloroquine resistance [5], [6] and evidence of *P. vivax* strains with lower sensitivity to primaquine [7], the development of a safe and affordable vaccine would be an important addition to control strategies for infectious diseases like malaria. Unfortunately, despite decades of research, effective malaria vaccines are still not available [8].

Several asexual blood-stage antigens have been identified as potential vaccine candidates and are strongly supported by evidence from studies using experimental models [9], [10]; immune recognition of these antigens has been demonstrated in exposed individuals in malaria-endemic areas [11], [12], [13], [14], [15], [16]. Among several vaccine candidates, the Apical membrane antigen 1 (AMA-1) is a well-characterized and functionally important merozoite protein and is currently considered to be a major candidate antigen for a malaria vaccine [17]. AMA-1 is a type I transmembrane protein that is expressed in the late asexual schizont stage of the *Plasmodium* parasite and accumulates in the micronemes of developing merozoites. Just prior to red blood cell (RBC) invasion, the mature form of AMA-1 is transported to the merozoite surface membrane as an 83 kDa protein in *P. falciparum*, or as a 66 kDa in other malaria species, where it is concentrated at the apical pole [18]; once on the surface, this form is redistributed and undergoes two C-terminal cleavages, giving rise to 48-kD and 44-kD soluble forms [19], [20], [21], [22]. AMA-1 is well-conserved in all *Plasmodium* species examined and is also present in other apicomplexan parasites [23], [24], [25], [26]. Although its precise role is still unknown, the crucial role of AMA-1 in the invasion process of *Plasmodium* species is clearly established [26], [27], [28], [29]. Several studies have demonstrated the association of AMA-1 with erythrocyte binding [30], [31] and reorientation of merozoites on the surface of red blood cells [29].

The AMA-1 ectodomain contains three distinct domains (I, II, and III) defined by eight intra-molecular disulfide bonds [32]. Several reports have previously shown that DII presents a high degree of amino acid sequence conservation [33], [34], [35] and is particularly the most immunogenic region of both *P. falciparum* [36] and *P. vivax* AMA-1 (PvAMA-1) [37] ectodomains. The determination of short and specific antigenic and/or immunogenic regions within vaccine candidates would represent an advantage over recombinant subunit vaccine development following the large-scale production and generation of chimeric peptides containing multiple relevant malarial epitopes.

In the present study, we describe the identification of a highly antigenic linear B cell epitope within the PvAMA-1 vaccine candidate and its further recognition in naturally *P. vivax*-infected individuals. We confirmed that DII presented similar antigenicity as the AMA-1 ectodomain, even when all IgG isotypes were evaluated. Our results add further support for the development of a PvAMA-1-based subunit vaccine against malaria vivax infection.

## Materials and Methods

### Study population and blood samples

A total of 214 blood samples from outclinic patients with uncomplicated *P. vivax* malaria were collected at endemic areas of Manaus (Amazonas state) and Cuiabá (Mato Grosso state). All patients were unrelated, as there were no family clusters, and they were attended to and diagnosed at the Fundação de Medicina Tropical Dr. Heitor Vieira Dourado, Manaus or Hospital Júlio Muller (Universidade Federal do Mato Grosso). Twenty healthy adult blood donors were recruited for the study over the course of several months from Belo Horizonte, Minas Gerais State, Brazil, a non-endemic area for malaria. The study was approved by the Ethical Committee on Research of Universidade Federal de Minas Gerais (Protocol# ETIC 060/07). Blood was obtained after receiving informed consent.

Venous blood was collected and was used to prepare thick smears for microscopy to extract parasite DNA and to perform serum separation. Parasitological evaluation was performed by examination of 200 fields at l.000× magnification under oil-immersion microscopy. All slides were examined by three well-trained microscopists from the Brazilian Ministry of Health. The *P. vivax* mono-infection was confirmed by PCR as previously described [38]. Infected individuals presented parasitemia levels of 3,726±9,624 parasites/µL. The mean age of the naturally infected and control groups were 38.6±14.0 and 36.4±11.9, respectively.

### Recombinant Pv-AMA-1 and Domain II of Pv-AMA-1 (DII)

The recombinant proteins representing amino acids 43 to 487 of Pv-AMA-1 (ectodomain) [39], [40] and the domain II (amino acids 249 to 385) of Pv-AMA-1 were expressed in *Escherichia coli*, as previously described [37], [39]. Briefly, the recombinant plasmid pHIS-AMA-1 or pET-28a-AMA-1-DII were transformed into *E. coli* BL-21 (DE3) expression host cells (Novagen, USA). Protein expression was obtained by inoculating 8 ml of a culture grown overnight in 200 ml of Luria broth (Invitrogen, USA) containing 100 µg/ml ampicillin (pHIS-AMA-1) or 30 µg/ml kanamycin (pET-28a-AMA-1-DII) (Sigma, USA). The culture was grown with continuous shaking at 37°C to an optical density of 0.6–0.8 at 600 nm and then induced for 3 hours under constant agitation at 37°C in the presence of 0.1 mM isopropyl-β-D-1-thiogalactopyranoside (IPTG, Invitrogen). The recombinant proteins were obtained from the pellet (ectodomain) or supernatant (domain II) of the bacterial lysates.

The pellet containing the PvAMA-1 ectodomain was processed for protein purification as described previously [37]. Briefly, the insoluble fraction, containing mostly inclusion bodies, was washed four times with 10 mM sodium phosphate buffer, pH 8.0, 1% 3-[(3-cholamidopropyl)-dimethyllammonio]-1-propanesulfonate (CHAPSO, Sigma). The washed inclusion bodies were then solubilized under continuous agitation in 10 mM sodium phosphate buffer, pH 8.0, 0.5 M NaCl, and 10% (vol/vol) glycerol containing 8 M urea at room temperature for 2 h. After centrifugation at 23,000 g at 4°C for 30 min, the supernatant was loaded onto a 1 ml column of pre-equilibrated Ni^2+^-NTA-agarose resin (Qiagen). The column was extensively washed with solubilization buffer containing 5 mM imidazole (Sigma) and then washed stepwise with decreasing concentrations of urea (6 M – 1 M) in refolding buffer (20 mM sodium phosphate buffer, pH 8.0, 0.5 M NaCl, 10% glycerol, 5 mM imidazole, 0.5 mM oxidized glutathione, 5 mM reduced glutathione, and 0.1% Triton X-100). Impurities were washed with five bed volumes of refolding buffer containing 80 mM imidazole, and PvAMA-1 was eluted with refolding buffer containing an imidazole gradient (0.1–0.4 M). The fractions were analyzed by SDS-PAGE and stained with coomassie blue. The fractions, containing highly purified recombinant protein, were extensively dialyzed against 50 mM sodium phosphate buffer, pH 8.0, 150 mM NaCl, 50% glycerol, and 0.1 mM DTT. The protein concentration was determined by the Bradford method (Bio-Rad) using bovine serum albumin (BSA, Sigma) as the standard.

The domain II of PvAMA-1 was purified as previously described [37]. Briefly, the soluble protein-containing supernatant was applied to a column with previously equilibrated Ni^2+^-NTA-agarose resin (sodium phosphate buffer 20 mM, pH 8.0, and 0.5 M NaCl). Bound proteins were eluted with a linear imidazole gradient (0.1–0.4 M) in wash buffer (sodium phosphate buffer 20 mM, pH 8.0, 0.5 M NaCl, 1 mM PMSF and 20% glycerin, pH 7.0). The fractions containing the recombinant proteins with a high degree of purity were pooled and dialyzed against 20 mM Tris-HCl, pH 8.0. After centrifugation at 18,000 g for 30 min at 4°C and filtration through a 0.22 µm membrane, the recombinant protein was purified by ion-exchange chromatography using an AKTA FPLC (GE Healthcare, Amersham, UK). The proteins were eluted with a linear 0–1 M NaCl gradient in Tris-HCl buffer. The 1 ml fractions were collected and analyzed by SDS PAGE. The fractions containing the recombinant proteins with a high degree of purity were pooled and extensively dialyzed against PBS. The protein concentration was determined as described above.

Both the ectodomain and domain II of the Pv-AMA-1 antigen were tested to determine the presence of Gram-negative bacterial endotoxin using a chromogenic Limulus Amebocyte Lysate test (QCL-1000, Cambrex, USA) according to the manufacturer's instruction and were found to be a non-significant source of endotoxins (levels lower than detection limit of 5 EU/mL).

### Sequence data

To predict possible antigenic properties in AMA-1 using bioinformatic tools, the entire PvAMA-1 sequence (Salvador strain, Accession Number AAC16731) was downloaded from the NCBI website (www.ncbi.nlm.nih.gov/protein). The signal peptide and the C-terminal portion of PvAMA-1 (corresponding to the transmembrane helix and intracellular portion) were manually removed in order to obtain the ectodomain sequence.

### Prediction of B cell epitopes

The prediction of B cell epitopes was carried out using the program BepiPred [41]. This software takes a single sequence in FASTA format as input, and each amino acid receives a prediction score based on known Hidden Markov Models profiles of known antigens and incorporates propensity scale methods based on hydrophilicity and secondary structure prediction. The lowest cut-off of 0.35 (as suggested by the BepiPred website) was used in at least nine consecutive amino acids in order to consider a given region as a valid linear B cell epitope. The epitope score represents the average of the scores of individual amino acids above the cut-off.

### Prediction of intrinsically unstructured/disordered proteins and domains

The prediction of intrinsically unstructured/disordered regions (IURs) was carried out using the program IUPred [42]. This software takes a single sequence in FASTA format as input and predicts the potential IURs. IURs do not possess sufficient inter-residue interactions to provide the stabilizing energy to overcome the entropy loss during folding, therefore, adopting loop structures. The final output is an individual score for each amino acid that ranges from 0 (completely ordered) to 1 (completely unordered). IURs were then predicted as a region spanning at least 9 contiguous amino acids with individual IUPred prediction score for each amino acid above 0.35. Bio::Graphics and in-house perl scripts were used to integrate the output of the BepiPred and IUPred prediction results.

### Synthesis, chromatography and mass spectrometry of soluble peptide

The peptide sequence SASDQPTQYEEEMTDYQK, corresponding to residues 290–307 of PvAMA-1 and having the best prediction scores for B cell epitopes and IURs, was manually synthesized by Fmoc chemistry [43]. The peptide was C-terminally amidated. The peptide was deprotected and released from the resin by TFA treatment in the presence of the appropriate scavengers. The peptide was lyophilized, and its purity and mass were assessed by HPLC and mass spectrometry, respectively.

The peptide was purified by high performance liquid chromatography (HPLC) in a C18 reverse phase column (Vydac) (flow rate 1.0 mL/min). The column was previously equilibrated with 0.1% aqueous trifluoroacetic acid (TFA), and the compound was eluted by a linear gradient of 0.1% TFA in acetonitrile. Two peaks were obtained (data not shown). The major peak was then submitted to MALDI-TOF-TOF analyses. MS and tandem MS analysis were performed using a MALDI-TOF-TOF AutoFlex III™ (Bruker Daltonics) instrument in positive/reflector mode controlled by the FlexControl™ software. Instrument calibration was achieved using Peptide Calibration Standard II (Bruker Daltonics) as a reference, and α-cyano-4-hydroxycinnamic acid was used as matrix. The major peak was spotted to MTP AnchorChip™ 400/384 (Bruker Daltonics) targets using standard protocols for the dried droplet method.

### Antibody measurement

The enzyme-linked immunobsorbent assay (ELISA) for total IgG antibodies was performed as previously described [39]. The concentrations of antigens used were 1.0 µg/mL (PvAMA-1 and DII) and 2.0 µg/mL (synthetic peptide). All samples were diluted 1∶100 and evaluated for total IgG using peroxidase-conjugated anti-human IgG antibodies (Sigma, St. Louis, MO). The ELISA to detect the IgG subclasses was performed as previously described [44]. The serum dilution used was 1∶100, and the mouse monoclonal antibodies to human IgG subclasses (clone HP-6012 for IgG1, clone HP-6014 for IgG2, clone HP-6010 for IgG3, and clone HP-6025 for IgG4, all from Sigma, USA) were diluted according to the manufacturer's specifications. Monoclonal antibody binding was detected with peroxidase-conjugated anti-mouse immunoglobulin (Sigma). The threshold of positivity (cut-off value) was obtained by testing 20 different negative control sera from individuals not exposed to malaria from Belo Horizonte. The mean optical density value at 492 nm±3 SD for duplicate determinations in negative sera was used as the cut-off value for different subclasses. The thresholds of positivity were 0.32 for IgG, 0.15 for IgG1, 0.13 for IgG2, 0.1 for IgG3, and 0.06 for IgG4. The reactivity index (RI) was obtained to compare the levels of different subclasses (IgG or IgG isotype) in the same subject or among distinct groups. The RI value was calculated by dividing the mean OD value for each test sample assayed by the cut-off value for each subclass tested using sera from healthy individuals (control group). Samples with an RI>1 were considered positive.

### Depletion ELISA

The depletion ELISAs were performed as previously described [45]. Briefly, flat-bottom plates (BD Falcon, USA) were coated overnight with 10 µg/mL of the peptide SASDQPTQYEEEMTDYQK, then washed, and blocked as described. Sera were added to the plates at a 1∶100 dilution and incubated overnight. On the following day, sera were transferred to plates coated overnight with PvAMA-1 and DII (1 µg/mL) after appropriate washing and blocking, and the ELISAs were performed as described.

### Statistical analysis

The one-sample Kolmogorov-Smirnoff test was used to determine whether a variable was normally distributed. All statistics were carried out using Prism 5.0 for Windows (GraphPad Software, Inc.). The association between antibody response to PvAMA-1 ectodomain and DII was determined by Fisher's exact test and Spearman rank test. P values for the depletion assays were also determined by Wilcoxon matched pairs test. A two-sided P value<0.05 was considered significant.

## Results

### Antibodies from naturally *P. vivax*-infected individuals present a similar pattern of recognition between the PvAMA-1 ectodomain and PvAMA-1 domain II

**Figure 1:**
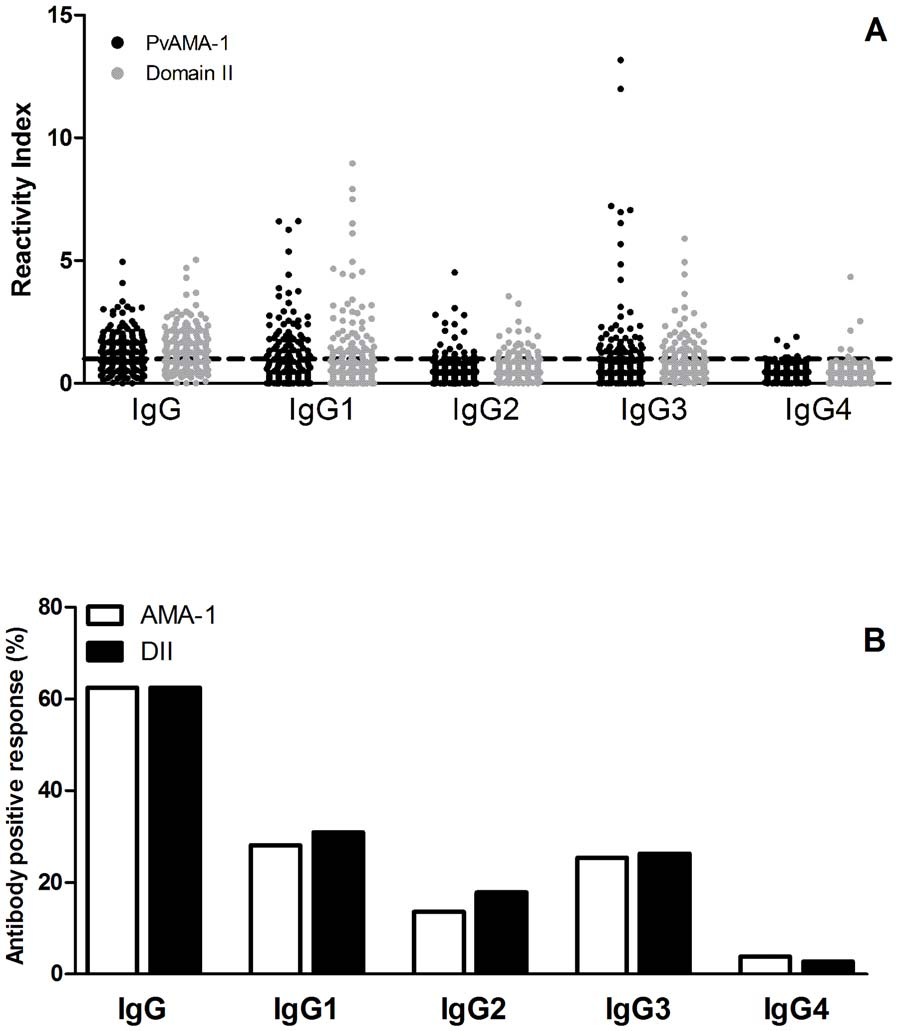
Levels and proportion of specific IgG and IgG subclass antibodies to PvAMA-1 ectodomain and PvAMA-1 Domain II (DII). (A) The Y-axis represents the mean reactivity index in naturally *P. vivax*-infected individuals. The dotted line shows the threshold of positivity (reactivity index = 1). (B) The Y-axis represents the mean prevalence of positivity observed in naturally *P. vivax*-infected individuals. Serum samples (n = 214) were tested at a 1∶100 dilution.

In order to confirm previously published data showing that the PvAMA-1 domain II is the immunodominant region of this protein, the detection of specific antibodies against both the PvAMA-1 ectodomain and DII was evaluated. Natural *P. vivax* infection elicits considerable IgG production against both PvAMA-1 ectodomain and DII antigens (Figure 1A). Although the levels of specific IgG1, IgG2, IgG3 and IgG4 against the AMA-1 ectodomain and domain DII varied in naturally *P. vivax*-infected individuals, the predominance of IgG1 and IgG3 antibodies to PvAMA-1 and DII was observed (Figure 1A).

**Figure 2:**
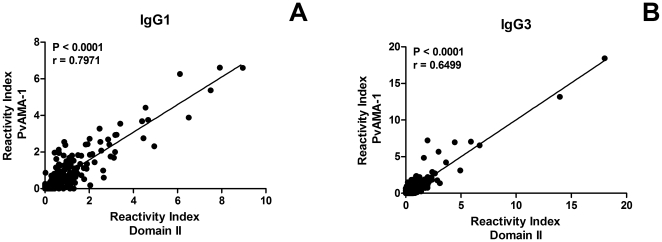
Specific antibodies to PvAMA-1 are directly correlated with antibody responses to Domain II. The correlation of PvAMA-1 reactivity index and Domain II reactivity index for (A) IgG1 and (B) IgG3 among 214 patients with *Plasmodium vivax* malaria was determined by Spearman rank correlation.

The overall prevalence of donors who presented IgG antibodies to each antigen was 62.4% (Figure 1B), although IgG-positive responders to each antigen were not necessarily the same (data not shown). Nevertheless, the frequency of IgG responders to the ectodomain was significantly associated with the frequency of IgG responders to DII (P<0.0418, Fisher's exact test). Analysis of specific IgG subclass production demonstrated that the association between individuals who responded to DII and responders to the full-length ectodomain was still significant (IgG1, P<0.0001; IgG2, P = 0.0002; IgG3, P<0.0001 and IgG4, P = 0.0007; Fisher's exact test), confirming that DII is a highly antigenic region within the PvAMA-1 protein. The strong association of specific IgG1 and IgG3 responses to the ectodomain and DII was further demonstrated by a direct correlation between the reactivity index for PvAMA-1 and the reactivity index for Domain II (P<0.0001, r = 0.7971 for IgG1 and <0.0001, r = 0.6499 for IgG3; Figure 2). A similar significant correlation was detected for IgG2 and IgG4 antibodies, although a minor relationship between the response to AMA-1 and DII was observed (data not shown).

### Predictions of B-cell linear epitopes and intrinsically unstructured/disordered regions in AMA-1 ectodomain and chemical synthesis of a selected peptide

**Figure 3:**
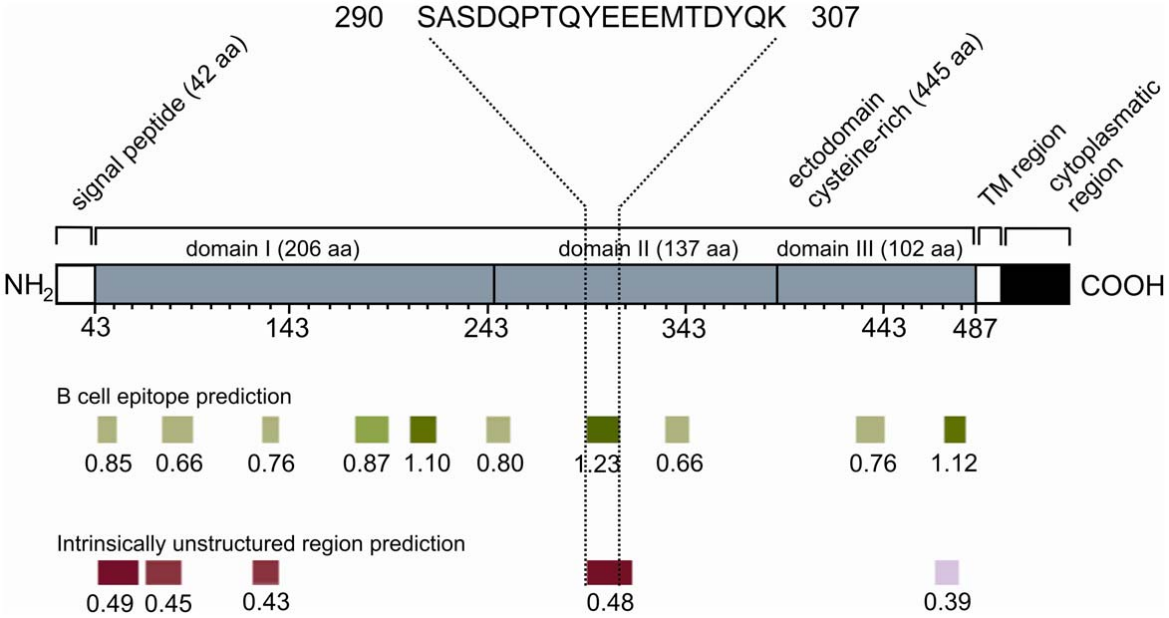
Schematic diagram of the structure of PvAMA-1, and the prediction scores for linear B cell epitopes and intrinsically unstructured/disordered regions (IURs). The region corresponding to the residues 290–307 of Pv-AMA-1 was selected for the synthesis of a soluble peptide based on the best prediction scores determined for both features. Each green bar represents a predicted B cell epitope, and each red bar represents a predicted unordered region. The prediction scores represents the average of scores for all amino acids within the region with prediction values above the cut-offs chosen for significance. The bar color intensities are proportional to the prediction scores found.

In order to explain the clear concordance between the IgG response to the ectodomain and DII, we scanned the primary sequence of AMA-1 ectodomain for possible B-cell linear epitopes co-occurring with intrinsically unstructured/disordered regions (IURs) (Figure 3). We observed several predicted epitopes distributed within all three domains of the ectodomain, with no apparent occurrence bias in any domain. Indeed, the three best predicted epitopes (5^th^, 7^th^ and 10^th^) are each distributed in a distinct domain (domains I, II and III, respectively). The putative IURs identified are also distributed in all three domains, with a slight preference for occurrence in domain I. Of special interest, however, was the overlap observed between the putative epitope possessing the highest prediction score (7^th^ epitope, score of 1.234) and the putative second best IUR (4^th^ IUR, score of 0.481) predicted in domain II. The other two best predicted epitopes (5^th^ and 10^th^) occur in regions where no IUR were found (5^th^) or in regions where IUR have a low prediction score (10^th^). Considering the higher prediction scores for both evaluated features, the peptide SASDQPTQYEEEMTDYQK was selected for further synthesis and immunological testing.

Analytical chromatography of the predicted peptide SASDQPTQYEEEMTDYQK demonstrated a purity of 80–90% for the synthetic compound (data not shown). Mass spectrometric analyses also indicated an estimated mass of 2150.017 Da that corresponded to the mass of the peptide (Figure S1).

### Synthetic malarial peptide SASDQPTQYEEEMTDYQK is highly recognized by specific antibodies to PvAMA-1 DII

**Figure 4:**
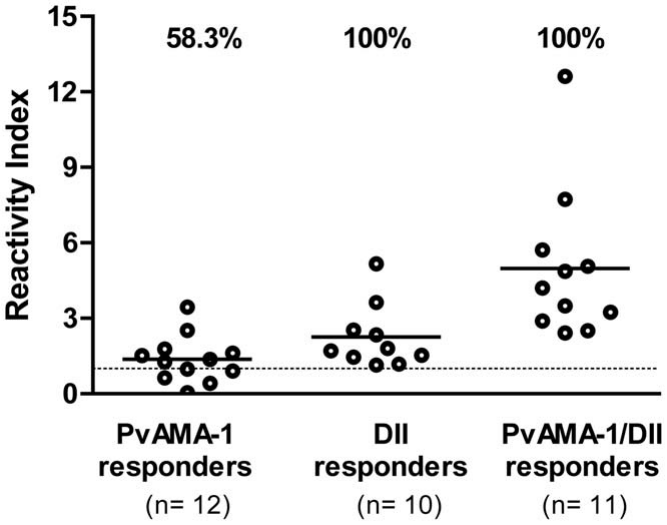
Levels of IgG against the synthetic peptide SASDQPTQYEEEMTDYQK. Serum samples were selected from naturally *P. vivax*-infected individuals who presented previous reactivity to PvAMA-1 ectodomain only, PvAMA-1 Domain II (DII) only or to both antigens. Antibody responses are expressed as the reactivity index detected by enzyme-linked immunosorbent assay. The horizontal dotted line shows the threshold of positivity at a reactivity index of 1. Percentage values represent the overall frequency of positive responders for each group.

**Figure 5:**
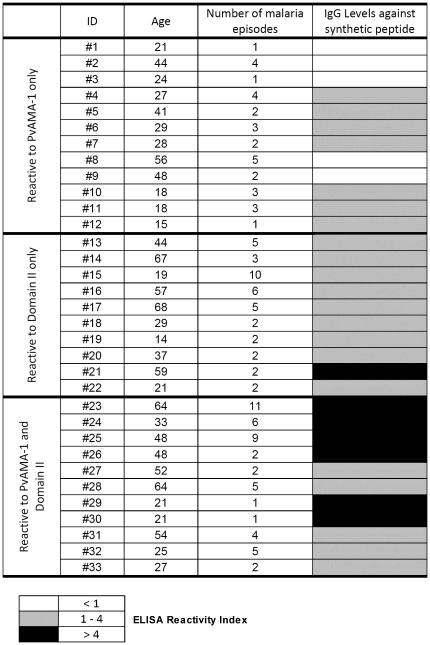
IgG antibody recognition of synthetic peptide in naturally *P. vivax*-infected individuals with known reactivity to PvAMA-1 ectodomain only, PvAMA-1 Domain II (DII) only or to both antigens. The age (years) and number of malaria episodes are indicated for each individual. Different levels of IgG antibody production (reactivity index) are represented with different shading patterns.

The antigenicity of the synthetic peptide SASDQPTQYEEEMTDYQK was initially evaluated by the determination of the specific IgG responses using sera from *P. vivax*-infected individuals who were knowingly reactive to the PvAMA-1 ectodomain only, domain II only, or reactive to both antigens. The data clearly show that the peptide was recognized by all serum samples that specifically recognized the domain II (Figure 4), thus confirming the previous prediction of B cell epitopes. When serum from infected individuals with no reactivity to DII (reactive only to PvAMA-1) was assayed against the synthetic peptide, a 58.3% positivity was observed (Figure 4), suggesting that the antibody production to this region in natural infection is present but that it is not sufficient to detect domain II. Of note, the highest reactivity indices for the synthetic peptide were observed in individuals that previously responded to both AMA-1 and domain II (Figure 5). No association between IgG levels against the synthetic peptide and exposure (determined by number of malaria episodes) was detected (Figure 5).

**Figure 6:**
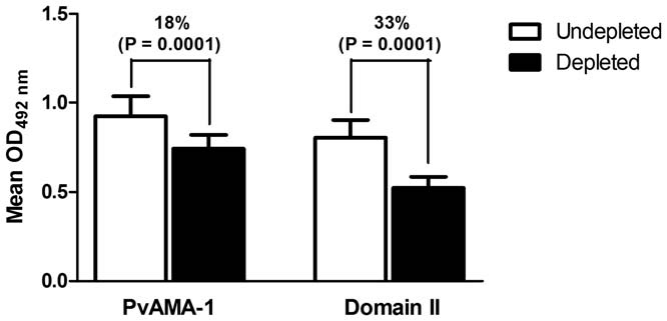
Immunodepletion results showing specific IgG antibody recognition to the synthetic peptide in naturally *P. vivax*-infected individuals with known reactivity to PvAMA-1 and Domain II (DII). The sera were depleted with the synthetic peptide SASDQPTQYEEEMTDYQK and further evaluated for specificity to PvAMA-1 and DII. The mean antibody OD values (n = 11) are shown on the Y-axis and the error bars indicate the SD. Statistical differences were detected using Wilcoxon matched pairs test and are indicated on the graphs with significant P values.

In order to determine the immunodominance of the synthetic peptide SASDQPTQYEEEMTDYQK within PvAMA-1 and DII antibody responses, an ELISA depletion assay was performed. In this assay, the IgG reactivity against PvAMA-1 and DII after depletion of peptide-specific antibodies was reduced by 18% and 33% (P = 0.0001 for both), respectively (Figure 6).

## Discussion

During the last decades, the development of subunit malaria vaccine candidates has been thoroughly pursued [46]. Although malaria vaccine development has been hastened by description of numerous candidates, no vaccine yet has provided a strong and lasting immune response [47]. Nonetheless, significant progress has been achieved as several malarial vaccines are currently undergoing pre-clinical and clinical trials [11], [46], [48]. Although more than 70 *P. falciparum* vaccine candidates are currently under development and 23 of theses are undergoing clinical testing, only a few *P. vivax* vaccine formulations are being assessed in pre-clinical studies, and so far, only two candidates (the CSP and the Pvs25) have been tested in Phase 1 clinical trials (reviewed in [49]). One of the most promising components for a malaria blood-stage vaccine is the apical membrane antigen 1 (AMA-1) due to the efficacy observed after a blood-stage challenge in animal models [11]. The observation of a protective outcome demonstrated that vaccination of mice and monkeys with native [50] or recombinant AMA-1 [10], [51] conferred protection from parasite challenge and induced antibodies that inhibit parasite growth *in vitro* [17], [40], [52]. These data have sparked interest toward the further clinical testing in humans; a few reports on Phase Ia trials in malaria-naïve subjects are already available for *P. falciparum* AMA-1 [53], [54], [55], and the results from ongoing and planned studies are expected in the near future for vivax malaria [17]. Although the success of this vaccine will be determined in field trials under natural conditions of parasite exposure, the continuous improvement of malaria vaccine design is still required. For instance, thus far, no recombinant malarial protein-in-adjuvant formulations have proved sufficiently efficacious to encourage further testing [56], [57]. Moreover, the production of high-quality and correctly folded *Plasmodium* recombinant proteins is difficult [58].

In the present study, our focus has been to characterize naturally antigenic B-cell determinants of the AMA-1 vaccine antigen in order to select future potential malarial subunit vaccines using bioinformatics tools and synthetic peptide technology. We initially confirmed that serum from naturally infected individuals used for further characterization of B cell epitopes was highly reactive to recombinant AMA-1 of *P. vivax*, where more than 60% of specific IgG recognition was detected among individuals with patent vivax malaria. In fact, immunoepidemiological studies have shown that specific antibody responses to AMA-1 are highly prevalent in individuals naturally exposed to malaria, even with varying levels of parasite exposure [14], [16], [39]. However, it is important to mention that due to the considerable sequence homology of AMA-1 among *Plasmodium* species [59], cross-reactive responses are expected to occur because *P. falciparum* is responsible for 16.3% of the registered malaria cases in Brazil [60]. We also demonstrated a similar proportion of IgG response between AMA-1 and domain II (DII) during natural infection, which corroborates a previous report showing that DII is a particularly antigenic and immunogenic region within AMA-1 [37]. Furthermore, the analysis of IgG subclasses indicated a predominant IgG1 and IgG3 antibody production against both AMA-1 and domain II. Of note, IgG1 and IgG3 antibodies have been implicated in antibody-mediated protective immunity against malaria [61]. Although the pattern of IgG subclasses against the recombinant AMA-1 has been clearly characterized in natural infection [14], [17], we are the first to demonstrate the prevalence of IgG isotypes specific to domain II in patent infected individuals. It is noteworthy that equivalent IgG subclass recognition between DII and the full AMA-1 ectodomain was also observed during natural infection. Interestingly, our analysis demonstrated that IgG and IgG subclasses elicited against AMA-1 are significantly associated with antibody production against DII.

The current understanding of AMA-1 immunity suggests that the direct action of antibodies is the primary protective mechanism [17]. Selection of parasitic epitopes that elicit protective antibodies after immunization would render a strategic approach in the design of effective vaccines. The recent availability of genomic [62], [63], proteomic [64] and transcriptomic [64], [65] data sets for *Plasmodium* species and the development of algorithms for B cell epitope prediction [41], [66] have facilitated the identification of promising vaccine antigens [67] and immunodominant regions within selected candidates. In the current study, we analyzed the primary sequence of PvAMA-1 for possible B cell linear epitopes co-occurring with intrinsically unstructured/disordered regions (IURs). Determinants containing both B cell linear epitopes and IURs are more likely to represent a real antigenic/immunogenic region within a protein. Indeed, the co-occurrence of IURs and linear epitopes increase the odds that the epitope is present in regions with no secondary structure (extended regions) that, therefore, are more likely to be exposed on the surface of the protein [68]. Our results showed that the prediction of B cell epitopes have identified antigenic regions distributed all over the three distinct domains of PvAMA-1, which might represent promising polypeptidic vaccine candidates. Considering the high degree of amino acid sequence conservation in the domain II loop in both *P. falciparum* and *P. vivax* AMA-1 [33], [34], [35], the strong association between AMA-1 and domain II antibody responses, and the epitope with highest prediction scores for both analyzed features was found in domain II, we have selected the peptide SASDQPTQYEEEMTDYQK for further chemical synthesis and immunological testing. Moreover, the analysis of this specific immunodominant region did not present homology to any genome sequence of mice and humans (data not shown), strengthening the rationale for its possible future use in pre-clinical and clinical trials.

Analysis of antigenicity of the selected peptide using serum from naturally *P. vivax*-infected individuals who were previously reactive to PvAMA-1 only, DII only or to both recombinant proteins demonstrated that the SASDQPTQYEEEMTDYQK peptide was recognized by all serum samples that also specifically recognized domain II. Moreover, serum from infected individuals with no reactivity to domain II presented a considerable positivity (58.3%) against the synthetic peptide, suggesting that during natural infection, there is specific antibody production against this antigenic epitope, which is probably not detectable when serum is assayed only against DII. Interestingly, although the number of malaria episodes has been associated with the frequency of antibody responses to PvAMA-1 in naturally infected individuals [39], our results did not show a clear association between IgG antibody recognition to the synthetic peptide and previous exposure to malaria. Finally, the results from the antibody depletion assay indicated that specific antibodies to the synthetic peptide would account for up to 18% and 33% of PvAMA-1 and domain II antibody responses, respectively. Overall, these results suggest that this AMA-1 linear epitope is highly antigenic during natural human infections, and it is an important antigenic region of the domain II of PvAMA-1. Although antibodies are widely agreed to play a role in protection against blood-stage malaria [56], the real contribution of the antibody response against this AMA-1 polypeptide remains to be addressed. Of note, a previous study establishing the epitope recognition by the invasion-inhibitory monoclonal antibody 4G2 specific for *P. falciparum* AMA-1 has demonstrated the importance not only of conformational epitopes but also of a singular disordered region with no electron density between residues 348 and 389 (equivalent to residues 293 to 334 in PvAMA-1) during parasite invasion [69].

In summary, in the current study, we have identified a highly antigenic B cell epitope within the PvAMA-1 vaccine candidate and its serological reactivity during natural infection. Selection of particular antigenic and immunogenic epitopes within the AMA-1 antigen would represent an interesting perspective to overcome the hurdles observed in vaccine development, mainly the production of high-quality and correctly folded proteins expressed heterologously, as a smaller antigenic polypeptide can facilitate large-scale production and generation of a chimeric peptide containing multiple relevant epitopes of malarial antigens. Pre-clinical studies are currently underway to determine whether the immunization with this peptide would elicit strong and lasting immunogenicity in mice as already demonstrated for the recombinant domain II [70].

## Supporting Information

Figure S1 **MALDI-TOF-TOF analysis of the synthetic peptide SASDQPTQYEEEMTDYQK.**
(TIF)
