# Supplementary figures and images for: Identification of a Highly Antigenic Linear B Cell Epitope within Plasmodium vivax Apical Membrane Antigen 1 (AMA-1)
Source: PLoS One. 2011 Jun 21;6(6):e21289. doi: 10.1371/journal.pone.0021289 (PMC3119695; doi:10.1371/journal.pone.0021289)

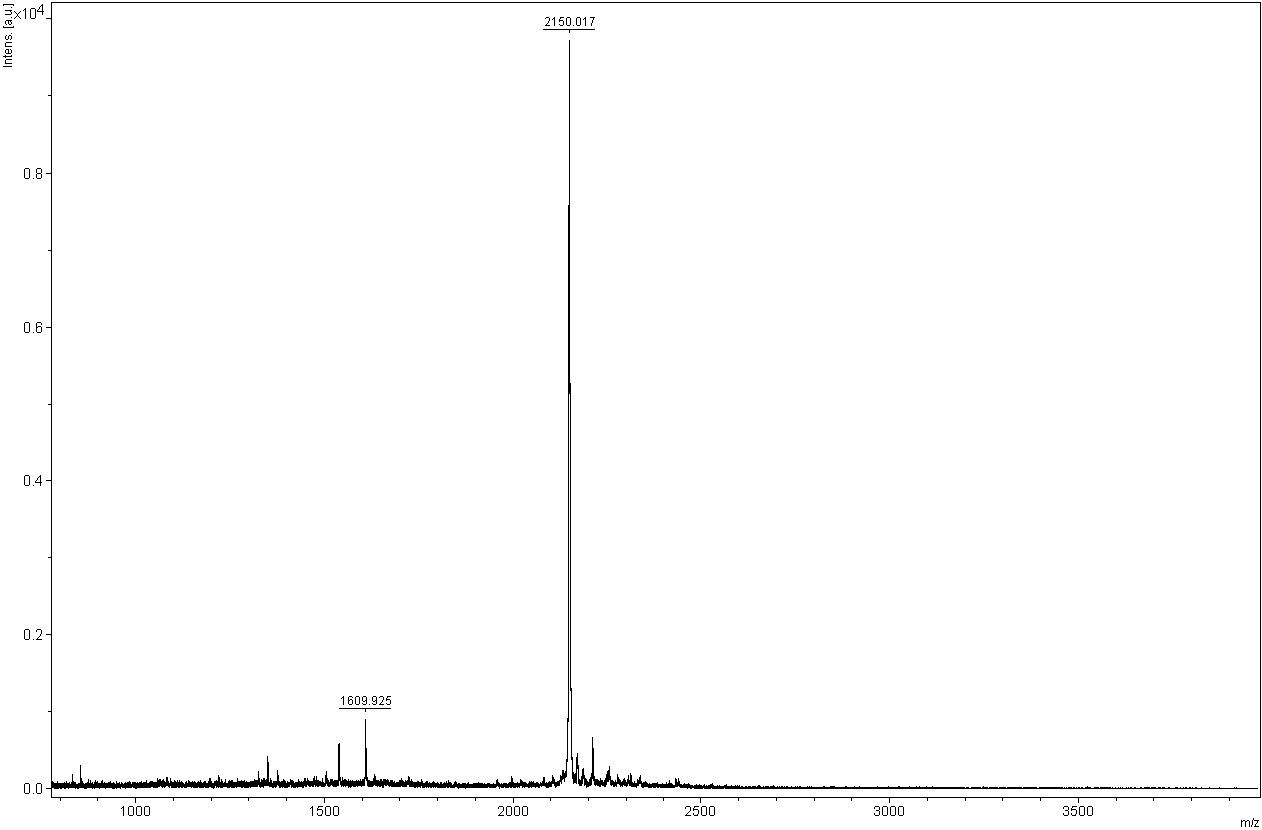

Supplement: Figure S1 — MALDI-TOF-TOF analysis of the synthetic peptide SASDQPTQYEEEMTDYQK. (TIF) [file pone.0021289.s001.tif]
